# Novel fructan exohydrolase: unique properties and applications for human health

**DOI:** 10.1093/jxb/ery268

**Published:** 2018-08-13

**Authors:** Wim Van den Ende

**Affiliations:** KU Leuven, Department of Biology, Leuven, Belgium

**Keywords:** Asparagus, fructan, fructan 1-exohydrolase, fructan 6G&1-exohydrolase, fructan exohydrolase, health, inulin neoseries-type fructan, inulin-type fructan, neokestose

## Abstract

This article comments on:

Ueno K, Sonoda T, Yoshida M, Shiomi N, Onodera S. 2018. Purification, characterization, and functional analysis of a novel 6G&1-FEH mainly hydrolyzing neokestose from asparagus. Journal of Experimental Botany **69,** 4295–4308.

**Fructans are multifunctional carbohydrates associated with stress responses in plants and microorganisms, and an array of well-characterized enzymes accounts for their wide diversity of structures in nature. However, a fructan exohydrolase enzyme involved in degrading the central β-2,6 linkage in neokestose and neokestose-based fructans has remained undetected. Ueno *et al.* (2018) not only filled this gap with the identification of a novel 6G&1-FEH from asparagus, but noted very special properties through its characterization. This will inspire further research into similar enzymes in other organisms, and open important new avenues for applied research.**

Fructans are water-soluble oligo- and polysaccharides mainly (with the exception of one glucose moiety) or exclusively consisting of fructose. ‘Classic’ fructans contain one internal or terminal glucose molecule, and are typically built from sucrose molecules. On the contrary, fructose-only type fructans, lacking glucose, originate from transfer reactions between these classic fructans and fructose or from endo-type enzymatic activity on classic fructans. Fructans occur in certain classes of Gram-positive and Gram-negative bacteria, some fungi and about 15% of flowering plants (Hendry, 1993; Toksoy Öner *et al.*, 2016).

Two types of linkages (either β ‐2,1 or β ‐2,6 or both) can occur between two adjacent fructosyl moieties. In addition, in some species a unique β ‐2,6 linkage can also occur between a fructosyl and the single glucosyl unit present (so-called neokestose-based fructans). This already leads to an enormous array of possible linear and branched variants, even more so because the degree of polymerization (DP) can differ greatly. Usually, very high DP fructans (>10000 fructosyl units) predominate in bacteria, although fructooligosaccharides (DP<10) may also occur. Importantly, in plants, overall DPs are much lower than in bacteria (usually<50, maximum DP ~300; Van den Ende, 2013). Bacteria and plants also differ greatly with respect to the location of fructan synthesis and operation (Kirtel *et al.*, 2018). Microbial fructans are synthesized from sucrose outside the cell and mainly function as extracellular polysaccharides in biofilms, although reserve functions and signalling functions for their breakdown products have been proposed (Kirtel *et al.*, 2018). Plant fructans are synthesized from vacuolar sucrose.

Besides their well-established function as a reserve polysaccharide, fructans are proposed to be multifunctional molecules during stress responses, acting as membrane and protein stabilizers, antioxidants and stress signals (Van den Ende, 2013). Inulin (β ‐2,1 linkages) is the most widely studied plant fructan and dominant in dicots such as chicory and Jerusalem artichoke, while levans (β ‐2,6 linkages) occur in grasses like *Dactylis glomerata* and *Phleum pratense* (Edelman and Jefford, 1968; Hendry, 1993). Graminans, branched-type fructans containing both β-2,1 and β-2,6 linkages, predominate in cereals like wheat and barley (Van den Ende *et al.*, 2003; Peukert *et al.*, 2014). Fructans that are built up from neokestose can be either neo-inulin or neo-levan type in plants. These neo-types occur in a long list of economically important species such as *Asparagus officinalis* (Shiomi, 1992), *Lolium perenne* (Pavis *et al.*, 2001) and onion (Vijn *et al.*, 1997), but also in wheat and durum wheat kernels at the milky stages (Verspreet *et al.*, 2013; Cimini *et al.*, 2015) and in *Agave* (Lopez *et al.*, 2003).

## Structural diversity of fructans in nature

Fructans are biosynthesized via the action of fructan biosynthesis enzymes or fructosyltransferases (FTs) which transfer a fructosyl unit from a donor substrate (either sucrose or a fructan) to an acceptor molecule (also either sucrose or a fructan). Bacterial FTs use a single enzyme mechanism to build their fructans, either by levansucrase or inulosucrase (Toksoy Öner *et al.*, 2016). These enzymes belong to the GH68 family of glycoside hydrolases. Plant FTs belong to the closely related GH32 family of glycoside hydrolases. Together, GH32 and GH68 comprise the GH-J clan, also containing plant cell wall and vacuolar invertases, as well as microbial invertases and endo-and exo-type fructanases (Versluys *et al.*, 2018).

Unlike microbes, plants synthesize fructans through the combined action of different types of FTs. Inulins are produced by the consecutive action of sucrose:sucrose 1-fructosyltransferase (1-SST) and fructan:fructan 1-fructosyltransferase (1-FFT). An additional branching enzyme, sucrose:fructan 6-fructosyltransferase (6-SFT) is required to synthesize cereal graminans, and a fructan:fructan 6G-fructosyltransferase (6G-FFT) is needed to create the unique β ‐2,6 linkage between fructose and the glucose part of sucrose in the neokestose molecule (also termed 6G-kestotriose) that forms the backbone for the neo-levan and neo-inulin type fructans (Versluys *et al.*, 2018).

Contrary to microbes, plants do not have fructan endohydrolases. Thus, fructan breakdown solely relies on the action of fructan exohydrolases (FEHs). Surprisingly, FEHs also occur in non-fructan accumulating plants such as *Beta vulgaris* and Arabidopsis, but their function remains puzzling (Le Roy *et al.*, 2013 and references therein). In fructan accumulators, fructan 1-exohydrolases (1-FEHs) and fructan 6-exohydrolases catalyse the breakdown of β-2,1 and β-2,6 linkages, respectively. The 6&1-FEHs are able to cleave both kind of linkages and 6-kestotriose exohydrolases (6-KEHs) specifically use 6-kestotriose as a substrate (Van den Ende *et al.*, 2005 and references therein).

The entire structural diversity of fructans in nature can be explained by build-up and breakdown activities of all the above-mentioned enzymes, and these enzymes were characterized and their genes cloned more than a decade ago. However, one very important exception remained: there was no enzyme reported able to efficiently attack the β ‐2,6 linkage between fructose and glucose in neokestose. This linkage forms a critical branching point that has to be specifically overcome, eventually allowing, in concert with other degradative enzymes, the complete hydrolysis of neo-inulin and neo-levan type fructans during remobilization processes (Box 1). The hunt for the missing enzyme, a presumptive 6G-FEH, was open for a very long time. Now, Ueno *et al.* (2018) have found this crucial enzyme in asparagus, a species accumulating both inulins and neo-type inulins.

**Box 1. Different plant fructan exohydrolases acting on selected basic fructan structures**

Sucrose, consisting of glucose (G) and fructose (F), forms the basis for fructan synthesis. A horizontal connection between two adjacent Fs signifies a β-2,1 linkage, while a vertical connection between two adjacent Fs signifies a β-2,6 linkage. The unique linkage between F and G in neokestose (6G-kestotriose) and neokestose-derived fructans is drawn at a different angle. 1-kestotriose is produced by 1-SST (purple) from two sucrose molecules and serves as donor substrate for neokestose synthesis by 6G-FFT (blue), as an acceptor substrate for branched bifurcose synthesis by 6-SFT (green) and as both donor and acceptor for inulin synthesis by 1-FFT (red). The latter enzyme is also involved in neo-inulin synthesis, while 6-SFT is involved in both neo-levan and levan synthesis, the latter being initiated by the intrinsic 6-SST activity of the 6-SFT. For simplicity, and to properly represent the position of FEH action, elongated versions of bifurcose and further elongation from the unique F2,6G linkage are omitted. The newly discovered 6G&1-FEH in asparagus (orange) suggests further research into possible 6G&6-FEHs acting on neo-levans (yellow) in other species. Since 6G&1-FEH does not degrade 1-kestotriose in asparagus, additional 1-KEH and/or 1-FEHs may be required to degrade this fructan species.

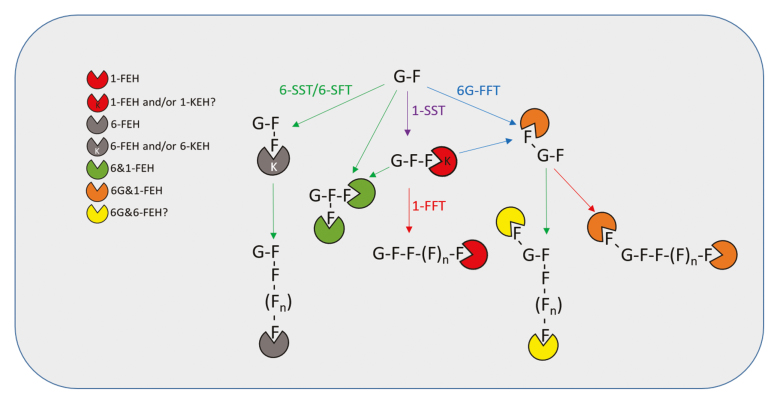

## Novel dimeric 6G&1-FEH with unique properties

How to hunt for a missing FEH that is central in neo-fructan degradation? First, choosing the right timing is essential, since some FEH isoforms may also act as ‘trimmers’ during fructan synthesis (Van den Ende *et al.*, 2003). Second, relying on sequence information derived from a genome is very tricky in terms of predicting possible functionalities within GH-J (Versluys *et al.*, 2018), and this is even more the case when hunting an enzyme with a new functionality. However, predicting which forms use sucrose as a donor substrate (invertase, SST) and which use fructan (FEH, FFT) can be done reasonably by looking at the presence or absence of the D/R couple in the hypervariable loop at the active site (Le Roy *et al.*, 2007). Besides 6G&1-FEH, Ueno *et al.* (2018) reported on other clones (aoeh1-3). Since aoeh3 contains a D/R couple, it probably represents a cell wall invertase.

Correlations between gene expression, enzyme activities and metabolite dynamics are sometimes absent for GH32 members (Lothier *et al.*, 2014 and references therein). Therefore, the best and most straightforward approach is not initiating the work at the DNA level, but purifying the native enzyme from an abundant source, in this case asparagus roots (Ueno *et al.*, 2018). In an elegant approach, the N-terminus of the purified native enzyme could be linked to one of the cloned genes, and expressing the latter in *Pichia pastoris* confirmed the novel 6G-FEH functionality.

This research revealed two big surprises: first, the same enzyme also showed intrinsic 1-FEH activity on inulins, but not on 1-kestotriose, being the smallest ‘classic’ inulin type fructan; and second, the enzyme exists in a dimeric form. The dual nature of the enzyme led to the term ‘6G&1-FEH’, which probably plays a central role in root inulin and neo-inulin type fructan degradation during the production of asparagus spears. It can be hypothesized that asparagus would need a dedicated 1-KEH acting in parallel with 6G&1-FEH. For the latter enzyme, it is likely that a completely different substrate-binding modus will be necessary to attack the β ‐2,6 linkage between fructose and glucose rather than for the hydrolysis of the β ‐2,1 bond between two fructose units. The fact that 1-kestotriose is a ‘bad’ substrate suggests that, in such configuration, the terminal glucose clashes with amino acids that reside in the vicinity of the active site, while this would not occur in the case of neokestose. The observation that longer inulins are a ‘good’ substrate suggests that some anchor points exist further away from the active site that help in stabilizing these longer chains. Another, and possibly more plausible explanation, is that evolution stimulated enzyme dimerization to reach this goal, with one enzyme unit assisting in substrate stabilization and the other one performing catalysis. To the best of my knowledge, dimerization has not yet been described within plant GH32 members, highlighting the unique nature of this special 6G&1-FEH enzyme. Examples of how dimerization and multimerization influence substrate specificity have been obtained for microbial GH-J members (Goldman *et al.*, 2008; Sainz-Polo *et al.*, 2013; Ramirez-Escudero *et al.*, 2016). 3D-structure analyses with bound ligands and structure–function mutagenesis will be necessary to test these intriguing hypotheses. Such insights may also contribute to resolving one of the greatest remaining mysteries when it comes to understanding structure–function relationships within GH-J members: which active site residues control the formation and degradation of β-2,6 versus β-2,1 linkages? Understanding these mechanisms would enormously boost rational enzyme design for the production of tailor-made fructans for use in food or non-food industrial applications (see also below). Furthermore, this novel 6G&1-FEH adds again to the enormous complexity of FEHs in fructan-accumulating plants. Different FEH classes should be discriminated in plants: (i) vacuolar FEHs, acting on endogenous vacuolar fructans; (ii) Type 1 apoplastic FEHs, acting on endogenous apoplastic fructans (cellular leakage, or transferred by stress-induced exocytosis); and (iii) Type 2 apoplastic FEHs, acting on fructan fragments of microbial origin, possibly acting as Microbial Associated Molecular Patterns (MAMPs; Versluys *et al.*, 2017). The endogenous apoplastic fructans have been considered as Damage Associated Molecular Patterns (DAMPs; Versluys *et al.*, 2017). The subcellular localization of 6G&1-FEH in asparagus requires further investigation.

Finally, the discovery of this unique plant enzyme attacking the β-2,6 linkage between fructose and glucose in neokestose and neokestose-based fructans raises another important question: do microorganisms contain similar enzymes? It can be speculated that microorganisms living in close interaction (either as a symbiont or pathogen) with neo-fructan accumulating plant species may have evolved such enzymes.

## Applications for asparagus production

Asparagus is a health-promoting (Ku *et al.*, 2018), fructan-accumulating vegetable with a commercial production estimated to be 190000 ha worldwide (Siomos, 2018). There are two main types of spears: green ones produced in the light and white ones produced in the dark (Siomos, 2018). In general, consumer interest in health-promoting vegetables is increasing year on year, and so further research into the mechanisms that may contribute to more-efficient asparagus production and/or better preservation of postharvest quality are required. Although the authors (Ueno *et al.*, 2018) demonstrated the up-regulation of 6G&1-FEH gene expression under cold storage, this was not associated with total fructan decreases. More in-depth analyses at the levels of protein amounts, enzyme activities and dynamics of fructan profiles (e.g. shift from higher to lower DP fructans) are required to understand whether the 6G&1-FEH enzyme plays a role during this process. During spear formation, 6G&1-FEH gene expression levels correlate well with total fructan decreases, suggesting its central role during inulin and neo-inulin remobilization from the roots. However, this also has to be corroborated by more thorough analyses. Additionally, previous studies on fructan dynamics and enzyme activities during postharvest storage of spears (Shiomi *et al.*, 2007) should be reconsidered and extended, in view of a possible central role for the newly discovered 6G&1-FEH, and links established to quality parameters. Finally, overexpression and CRISPR/Cas deletion approaches on asparagus 6G&1-FEH would provide an excellent tool for studying the relative importance of this gene during the production and subsequent storage of asparagus spears.

## Controlling regrowth and postharvest sprouting

Fructan-accumulating fodder grasses greatly contribute to milk and meat production, and therefore these are subject to intense investigation. *Lolium perenne* is probably the most thoroughly investigated grass in this respect. Contrary to asparagus, neo-levan type fructans predominate, and these typically accumulate to a great extent in the lower parts of the plant, just above the soil surface. After grazing or artificial mowing, these fructans are rapidly remobilized by increasing total FEH activities, but this paradoxically coincides with decreasing gene expression levels for 1-FEH and 6-FEH (Lothier *et al.*, 2014). One possible explanation is that FEHs are already present at higher levels before defoliation, but kept inactive by sucrose occupying the active site as an inhibitor (Verhaest *et al.*, 2007). After removal of the photosynthetic apparatus, sucrose levels moving downwards through the stems and entering the lower parts would greatly decrease, leading to a new FEH/sucrose equilibrium that releases the sucrose from the active site and activates the FEHs, resulting in prompt fructan degradation to sustain rapid leaf regrowth. Another, or additional, explanation that would overcome the above-mentioned paradox is the presence of a yet unidentified FEH that would be transcriptionally up-regulated after defoliation. In this sense, the discovery of asparagus 6G&1-FEH should inspire the fructan community to reconsider this matter, as it hints at the existence of one or more possible novel 6G&6-FEH enzymes, besides 6G&1-FEHs (Box 1), operating in perennial ryegrass. Just as sugar signals (e.g. T6P) and the SnRK1/TOR/autophagy network (Soto-Burgos and Bassham, 2017) are involved in controlling the balance between starch and small soluble sugars, mainly by regulating amylases (Santelia and Lunn, 2017 and references therein), similar mechanisms may occur during fructan remobilization controlled by FEHs (Lothier *et al.*, 2010). Fundamental knowledge on these connections could pave the way to slow down grass regrowth in lawns, a highly desired trait for many people, or further speed up grass regrowth in terms of feed production.

Needless to say, the way is now open for exploring the presence of 6G&1-FEH and/or 6G&6-FEH type of enzymes in other neo-fructan accumulating plants such as onion, *Agave*, oat and wheat kernels, among others. If it turns out that these enzymes are main players in overall neo-fructan remobilization, their inhibition could greatly contribute to practical applications. For instance, suppression of onion sprouting is highly desired, since high costs and environmental drawbacks are associated with long-term cold storage and/or the use of expensive and sometimes toxic chemicals to prevent sprouting (Petropoulos *et al.*, 2016).

## Changing fructan contents for human health

Inulin-type fructans are well recognized health improving compounds acting as prebiotics (indirect effects on gut microbiota) and/or immunomodulatory agents, stimulating the native immune systems of animals, humans and plants in responses against pathogens. This might decrease the use of antibiotics and toxic agrochemicals, which is of high socio-economical value (Peshev and Van den Ende, 2014 and references therein). The exploitation of other fructan types (graminans, agavins, levans) is in its infancy and the inclusion of fructans in a wider array of basic food products is expected (e.g. fructan bread, fructan cheese). Also, genome editing programs on FTs are expected to lead to fructan-enriched grains and fruits. However, overdosing on fructans may lead to negative effects in sensitive subjects, especially those suffering from inflammatory bowel disease (IBD), and it would also be interesting to develop products with decreased fructan content for them (Struyf *et al.*, 2017 and references therein). Wheat is by far the most important fructan source in the Western diet, accounting for ~70% of the daily fructan intake (Moshfegh *et al.*, 1999). The proportion of neo-type fructan structures in the total fructan pool gradually increases during wheat grain development, leaving neokestose, 6G,6-kestotetraose, 6G,1-kestotetraose and 1&6G-kestotetraose as major components in wheat flour (Verspreet *et al.*, 2017). In turn, this suggests that the natural fructan degradation machinery in wheat kernels is not very efficient at hydrolysing the β ‐2,6 linkage between fructose and glucose in neokestose-based compounds. Certainly, further research into 6G-FFT, 6G&1-FEH and 6G&6-FEH enzymes in wheat kernels is needed. Genome-editing programs may lead to a fine control of these compounds in wheat flours. In addition, recombinant 6G&1-FEH and 6G&6-FEH enzymes may be added during food processing (e.g. breadmaking) to lower fructan levels in food for IBD patients.
